# Diamidophosphate (DAP): A Plausible Prebiotic Phosphorylating Reagent with a Chem to BioChem Potential?

**DOI:** 10.1002/cbic.202100274

**Published:** 2021-08-08

**Authors:** Abdulakeem Osumah, Ramanarayanan Krishnamurthy

**Affiliations:** [a] Department of Chemistry, The Scripps Research Institute, 10550 North Torrey Pines Rd, La Jolla, CA 92037 (USA)

**Keywords:** cyclophosphate, diamidophosphate, origins of Life, phosphorylation, prebiotic chemistry

## Abstract

Known since the 1890s, diamidophosphate (DAP) has been investigated within the context of its inorganic chemistry. In 1999 – with the demonstration of DAP’s potential as a phosphorylating agent of sugars in aqueous medium – began the exciting phase of research about DAP’s role as a plausible prebiotic phosphorylating agent. More recently, in the last five years, there has been a steady increase in the publications that have documented the surprising versatility of DAP enabling the emergence of many classes of biomolecules of life, such as nucleic acids, peptides and protocells. Thus, though in its infancy, DAP seems to be uniquely positioned to play a central role in modelling abiotic- to prebiotic-chemical evolution. In this context, there is a need for systematic investigations for: (a) establishing DAP’s likely availability on the early Earth, and (b) developing DAP’s potential as a tool for use in synthetic and bioorganic chemistry.

## Graphical Abstract

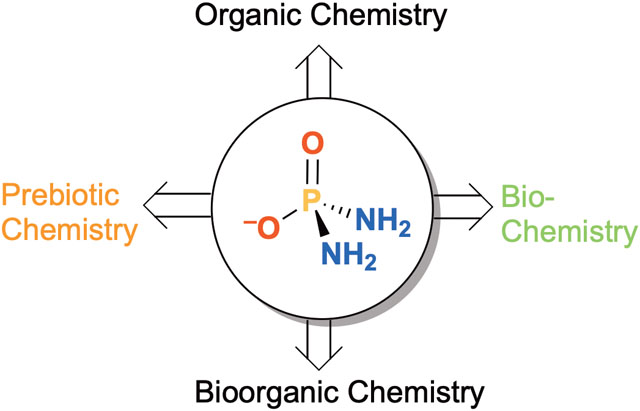

**Diamidophosphate (DAP)**, a di-nitrogenous analogue of orthophosphate, is a versatile phosphorylating-activating reagent and amine source in the context of aqueous prebiotic chemistry compatible with both prebiotic- and bio-molecules. The promising potential of this reagent, enabling a continuous transition from abiotic-to-biotic needs to be investigated both from its geochemical provenance and its applicability to (bio)organic transformations.

## 1. Diamidophosphate (DAP): A Historical Background

### 1.1. Synthesis

**Scheme 1.**
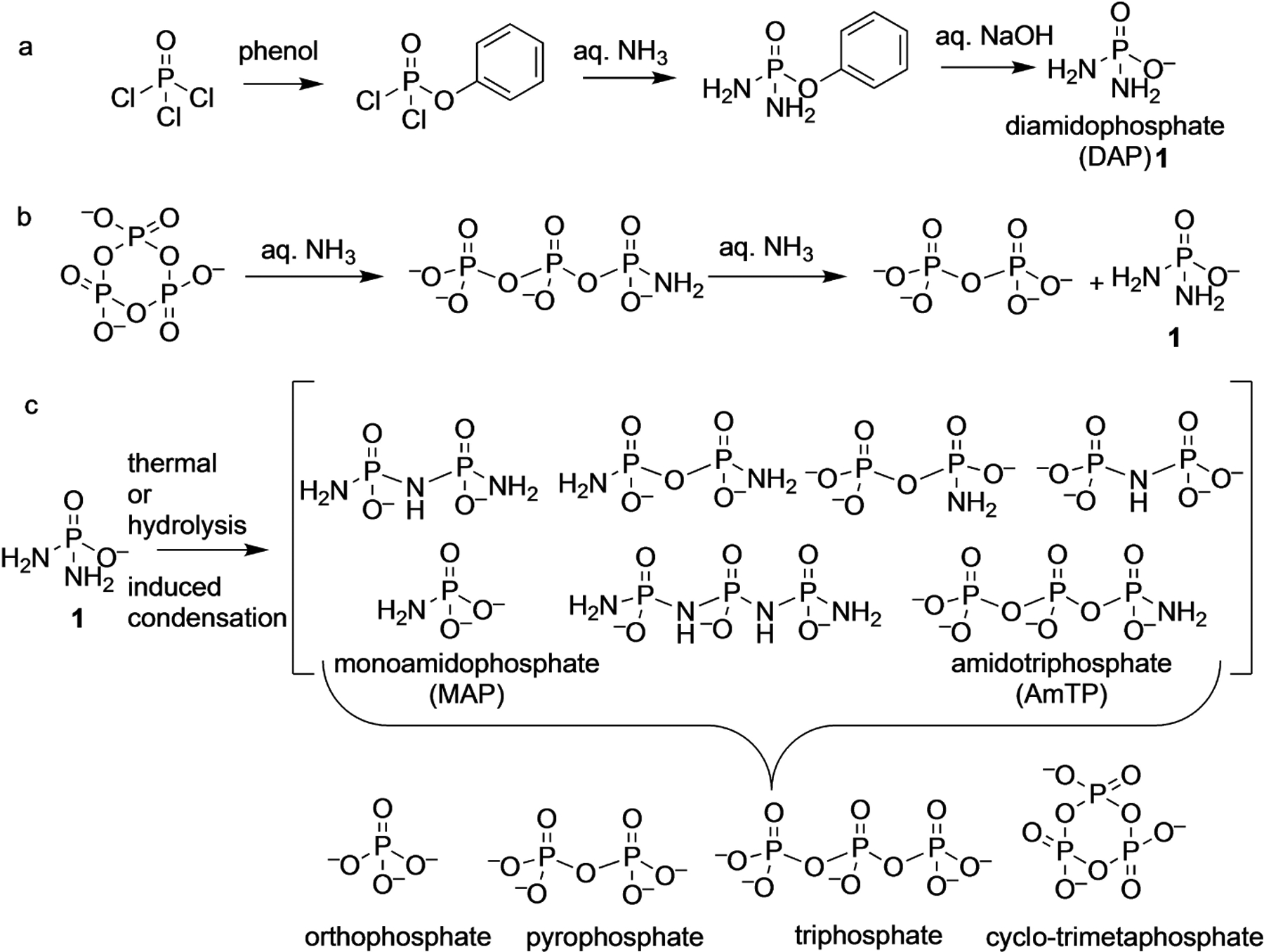
(a) Synthetic preparation of DAP. (b) Formation of DAP by ammonolysis of cyclic-trimetaphosphate. (c) Thermal and hydrolysis induced reaction of DAP leads to phosphoramidate, phosphoroimidate and phosphate species, many of which are themselves good phosphorylating agents.

Diamidophosphate (DAP, **1**) was first reported by Stokes in 1894, as part of a series of reports on amidophosphoric acids, outlining its preparation and of its various salts, by ammonolysis of phenyldichlorophosphate followed by hydrolysis of the phenylester (Scheme 1a).^[1]^ Since then, with an aim to improve the yield, the synthesis of DAP was revisited which led to various routes, with phosphorus oxychloride (POCl_3_) as the predominant starting material.^[2]^ For example, hydrolysis of phosphoryltriamide, which itself is prepared from POCl_3_, was found to produce DAP. Instead of POCl_3_, condensed phosphates (e.g., trimetaphosphate) when reacted with ammonia for prolonged time produced DAP (Scheme 1b),^[3]^ though this method has not been used for preparative purposes. Hydrolysis of condensed imido-polyphosphates have been shown to form DAP.^[4]^ The ammonolysis of triphosphorus pentanitride, P_3_N_5_, under high temperature and pressure leads to crystalline DAP.^[5]^ Currently, the original procedure developed by Stokes^[1]^ (via saponification of phenyl phosphorodiamidate), with subsequent modifications,^[6]^ seems to be the best method for preparing DAP in large quantities (Scheme 1a).

### 1.2. Properties and reactions

#### 1.2.1. Hydrolysis of DAP

The chemical properties of various salts of DAP, especially in terms of their hydrolysis and thermal behavior have been studied extensively.^[7]^ In general, DAP (as is with amidophosphates in general) was found to undergo loss of ammonia to form a spectrum of condensed products containing P–O–P and/or P–N–P bonds, with orthophosphate as the stable end-product (Scheme 1c).^[8]^ Importantly, DAP forms condensed polyphosphate species in water without the need for activating or condensing reagents, which makes it potentially attractive from a prebiotic (and biotic) phosphorylating and activation viewpoint.^[9]^

Although DAP is monoanionic, two values of $\text{p}K_{a_{1}}\;\approx \;1.2$ and $\text{p}K_{a_{2}}\;\approx \;5.0$ have been observed; $\text{p}K_{a_{2}}$ is assigned to the protonation for one of the NH_2_ groups, while $\text{p}K_{a_{1}}$ assignment was left ambiguous.^[10] $\text{p}K_{a_{1}}$^ likely involves the second amino group as seen from the sequential hydrolysis of DAP to orthophosphate with the involvement of monoamidophosphate (MAP) as an intermediate (Scheme 1c). This disparity between the two amino groups in DAP and the close-to-neutral $\text{p}K_{a_{2}}$ enables the kinetic stability towards hydrolysis at near neutral pH while allowing for a controlled reactivity of DAP as a phosphorylating agent.^[7]^

Up to the late 1990s, study of the reactions of DAP remained within the realm of inorganic chemistry as outlined above, despite a plethora of work since the early 1950s showing the importance of organic-amidophosphate derivatives in many research areas.^[11]^ Of relevance to this review are the phosphor-oamidates of biological molecules which were synthesized by condensation of ammonia with already phosphorylated derivatives, such as phosphoramidate derivatives of nucleosides and oligonucleotides.^[12]^

## 2. DAP: Reactions with Organic Substrates

Phosphorylation is an important process in biochemistry. Most biological molecules contain phosphate groups (nucleic acids, phospholipids, and metabolites), and hydrolysis of phosphate bonds (e.g., ATP) provide energy for biological processes.^[13]^ Therefore, understanding how inorganic phosphates became a part of biology has become central to understanding the chemical origins of life’s processes.^[14]^ Thus, abiotic phosphorylation of prebiotically plausible organic molecules became an active area of study.^[15]^ This section describes how the initial discovery of DAP’s role in prebiotic phosphorylation has led to continuing investigations of its reaction in a *Chem*(istry) and *Biochem*(istry) context.

### 2.1. Prebiotic phosphorylation

Prebiotic phosphorylation of biologically relevant molecules is being investigated primarily (still) with orthophosphate since it is the most widely available P-species on early Earth.^[15,16]^ However, orthophosphate is unreactive, and robust activation processes and/or non-aqueous conditions are employed for the initial step of introducing the phosphate,^[17]^ which are incompatible for the next steps of producing the corresponding oligomers and functional higher-order biological structures. Thus, spatially separated processes and different activating mechanisms need to be invoked at different stages.^[16]^ This has led to the consideration of alternate phosphorylating agents, such as naturally available reduced phosphorus species, for example, phosphites.^[18]^

**Scheme 2.**
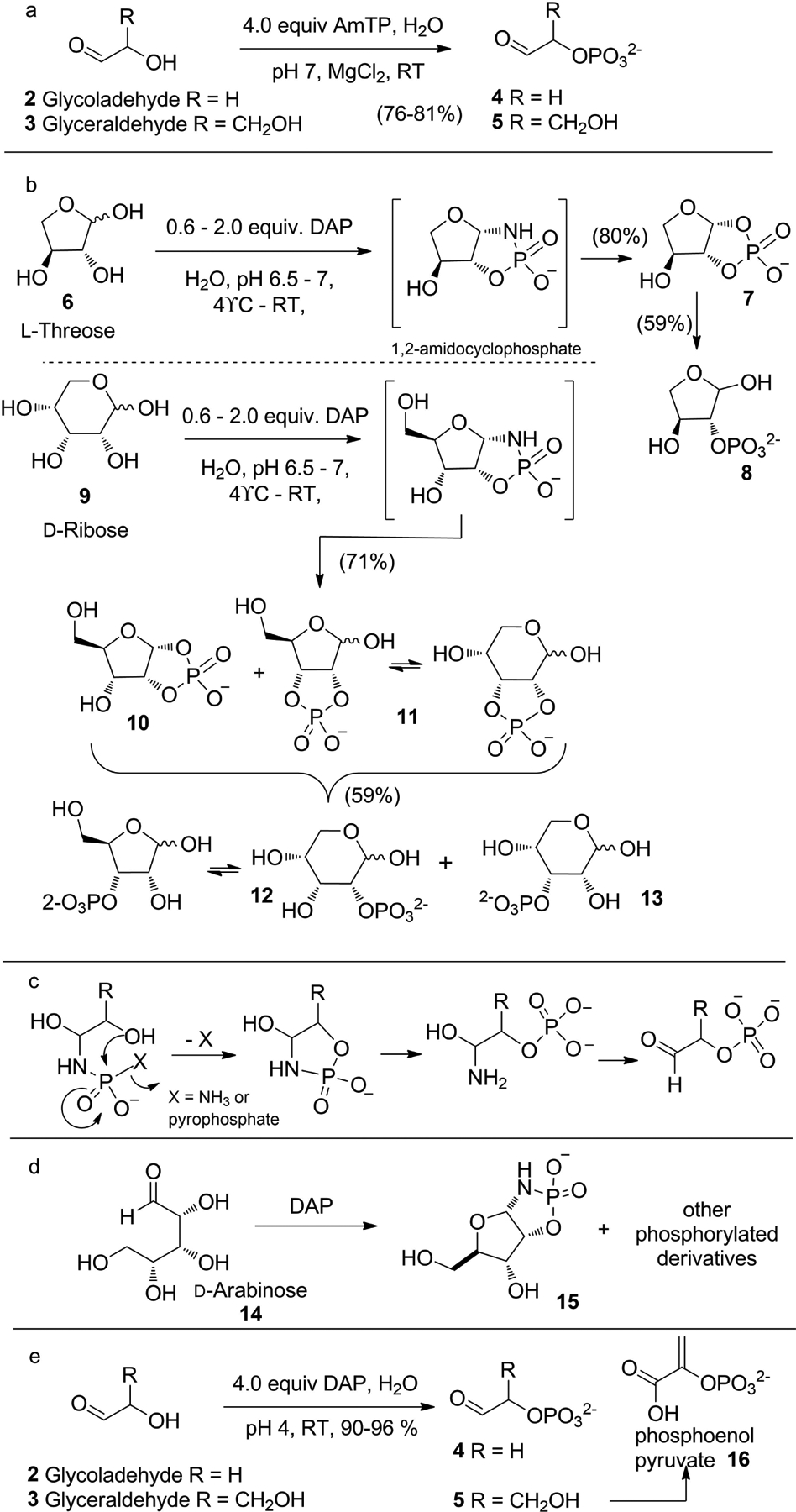
AmTP and DAP-mediated regioselective (intramolecular) phosphorylation of α-hydroxyl aldehydes (aldoses) in water.

In 1999, a different paradigm of using PN compounds for prebiotic phosphorylation in aqueous medium, emerged with the report from Krishnamurthy et al. where amidotriphosphate (AmTP) and diamidophosphate (DAP) were shown to phosphorylate various α-hydroxy-aldehydes and aldose-sugars efficiently in water (Scheme 2).^[19]^ The inspiration to use DAP was derived from the use of AmTP, which phosphorylated the acyclic aldoses, glycolaldehyde **2** and glyceraldehyde **3** converting them to the corresponding α-phosphates, glycolaldehyde phosphate **4** and glyceraldehyde phosphate **5** respectively (Scheme 2a).^[19,20]^ Threose **6** and ribose **9** were converted to predominantly the furanose-1,2-cyclophosphates **7** and **10** respectively via their furanose-1,2-amidocyclophosphate intermediates. 2,3-Cyclophosphate derivative **11** of ribose (in equilibrium with the furanose and pyranose form) was also formed, and all these cyclophosphate compounds could be hydrolyzed to their corresponding monophosphate derivatives **8**, **12** and **13** (Scheme 2b). The efficient regiospecific phosphorylation of the α-hydroxy groups of aldoses by AmTP and DAP is mechanistically different from the previous intermolecular water-elimination process. The nucleophilic attack of the NH_2_ group of DAP (or AmTP) on the carbonyl group tethers the amidophosphate close to the α-hydroxy group (Scheme 2c), which facilitates an intramolecular phosphate transfer with the elimination of NH_3_ (or pyrophosphate). The amido-phosphorylation enabled synthesis of 1,2-cyclophosphates of ribose and threose has been successfully employed by Benner, Kim and coworkers in their demonstrations of prebiotic synthesis of canonical and non-canonical nucleotides.^[21]^

In 2008, Sutherland and co-workers harnessed the phosphorylation potential of DAP by reacting it with arabinose **14** forming the corresponding cyclic phosphoramidate **15** and cyclophosphate products along with other phosphorylated derivatives of arabinose (Scheme 2d).^[22]^ The next report was in 2017 by Powner and coworkers, where they used DAP to phosphorylate the α-hydroxyl groups of glycolaldehyde **2** and glyceraldehyde **3** which served as substrates for the prebiotic synthesis of phosphoenol pyruvate (Scheme 2e).^[23]^ All of the above reactions reinforced the original mechanism^[19]^ and implied that the intramolecular-phosphate-transfer by DAP will be substrate-limited to reactive aldehyde-containing compounds (Scheme 2c).

**Scheme 3.**
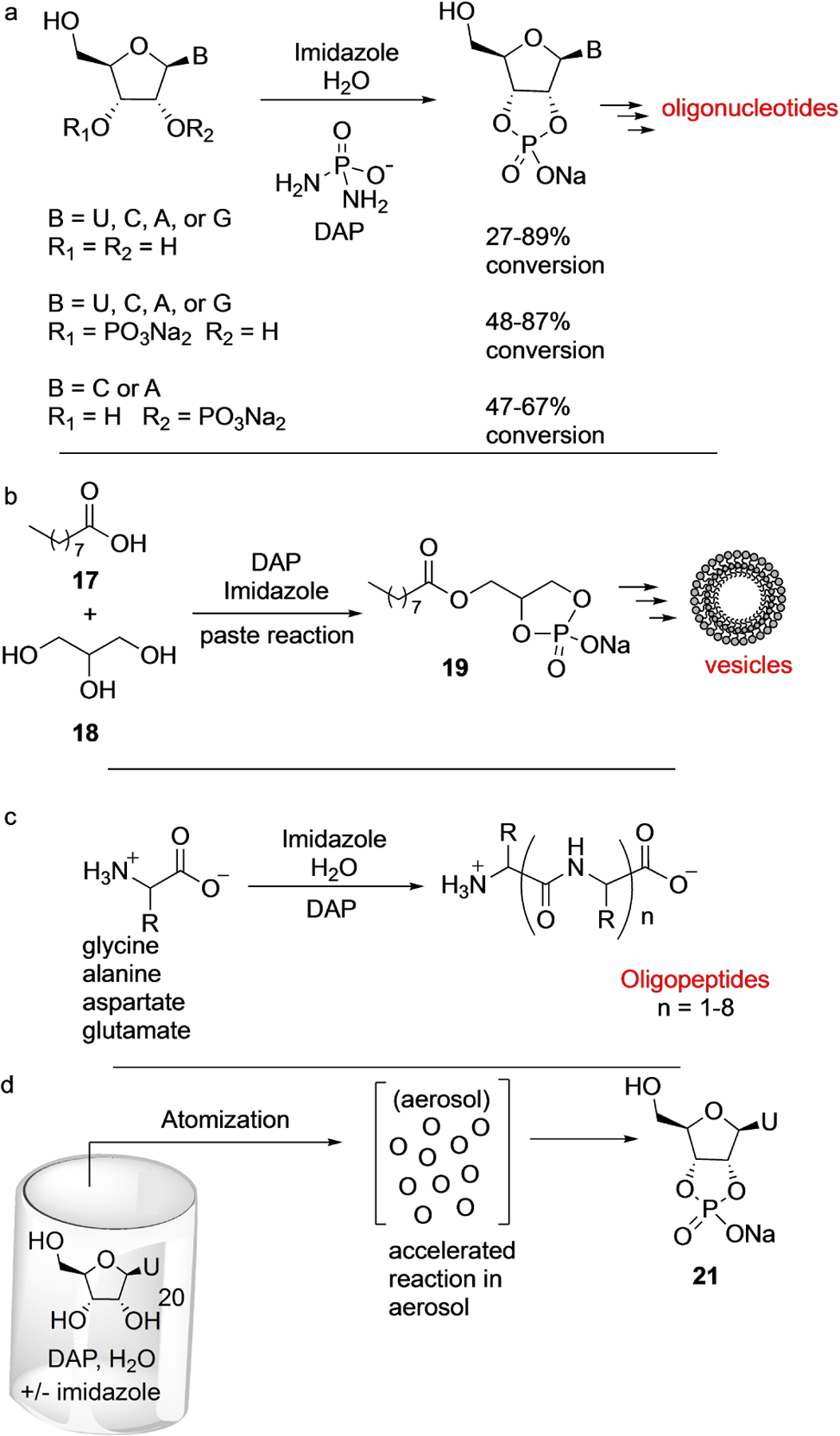
DAP-mediated Prebiotic phosphorylation of (a) nucleosides and nucleotides in aqueous medium forming cyclophosphates, (b) mixtures of glycerol/fatty acids under moist-paste conditions which lead to the formation of vesicles, (c) amino acids which give rise to short peptides. (d) Under aerosol condition DAP-mediated phosphorylation of uridine is highly accelerated (within hours) when compared to the bulk solution (days-weeks). A=adenine, C=cytosine, G=guanine, and U=uracil.

This perception, however, had to be revised based on the results reported by Gibard et al. in 2018,^[9]^ who showed that DAP is able to phosphorylate hydroxyl groups that lack the aldehyde moiety (Scheme 3). This was born from the observation that DAP hydrolysis in water leads to polyphosphates, thus opening the possibility that the NH_2_ group in DAP can be replaced by other nucleophiles intermolecularly.^[9]^ DAP was shown, in the presence of imidazole and divalent metals as catalysts, to phosphorylate ribonucleosides, amino acids, and glycerol in water over a range of pH values and temperatures. The DAP-mediated phosphorylation reactions were slow and took place over days/weeks. Ribonucleosides formed the corresponding 2′,3′-cyclophosphates in good yields, while glycerol formed 1,2-cyclophosphate (Scheme 3a,b). When DAP-phosphorylation was performed under low water-activity (‘moist-paste’) conditions at room temperature, pyrimidine ribonucleoside-2′,3′-cyclophosphates were formed in good yields, but purine ribonucleoside-2′,3′-cyclophosphates were obtained in poor yields. Unexpectedly, oligomers were observed in the case of uridine, which suggested that DAP was also acting as an activating agent under the same reaction conditions and converting the thus formed nucleotides to oligonucleotides. Amino acids (glycine, alanine, aspartic and glutamic acids) were phosphorylated both at the α-amino and α-carboxylate moieties that led to oligomerization forming short peptides in aqueous medium (Scheme 3c). Parker et al. showed that introduction of wet-dry cycles increased the efficiency of peptide bond formation.^[24]^

Furthermore, when glycerol **18** and long-chain fatty non-anoic acid **17**, DAP, and imidazole were reacted, the resulting mixture formed many species, among them cyclophosphatidic acid **19** (Scheme 3b). And when this crude reaction-mixture (or pure **19**) was hydrated, it led to the formation of vesicles.^[9]^ Later, Duhan et al. in 2019 showed that indeed these cyclophospholipids increase the stability of fatty acid protocells.^[25]^ Thus, phosphorylation by DAP of the three different classes of molecules, nucleosides, amino acids, and glycerol/fatty acids -under similar ambient conditions- led to their corresponding higher-order structures, oligonucleotides, peptides, and protocells, respectively.

Castañeda et al.^[26]^ investigated the effect of aerosol environment (to mimic the early Earth ocean-air interface) on DAP-mediated phosphorylation of uridine **20** and observed rapid phosphorylation of uridine within hours to form the corresponding 2′,3′-cyclophosphate **21** (Scheme 3d). The dramatic increase in the rate of reaction was attributed to (a) the higher concentration and closer proximity of reagents, (b) imidazole activation of DAP, (c) longer residence time of droplet particles in the aerosol chamber, and (d) the larger surface-area-to-volume ratio of the aerosols. This approach to mitigating the unfavorable rates of DAP phosphorylation in bulk solution in aerosol environments, in principle, could be applied to the phosphorylation of other classes of molecules.

**Scheme 4.**
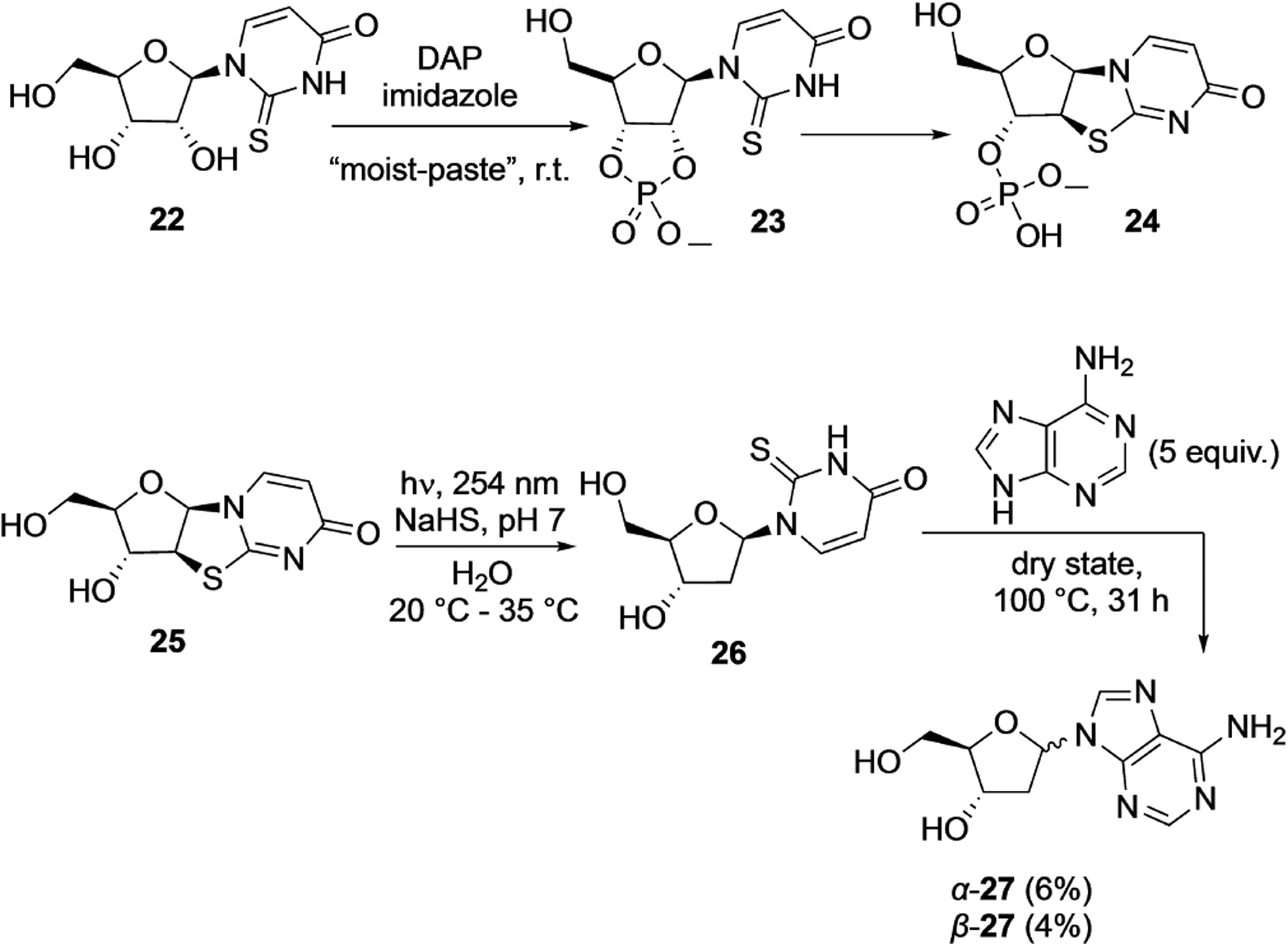
Application of DAP-mediated cyclophosphorylation for prebiotic synthesis of deoxynucleosides. Adapted from Ref. [27].

The Sutherland group in 2019^[27]^ applied this DAP-mediated cyclophosphosphate activation in their prebiotic synthesis of deoxynucleosides from pyrimidine-ribonucleosides (Scheme 4). For example, DAP reaction with 2-thiouridine **22** generated 2-thiouridine 2′,3′-cyclophosphate **23**, which proceeds under the same reaction conditions to form the 2-thioanhydride derivative **24**. Light mediated reduction of dephosphorylated derivative **25** in the presence of aqueous H_2_S led to the formation of deoxyribose derivative **26** which was used to generate deoxyribose in situ and trapped by adenine to form deoxyadenosine **27**.

**Scheme 5.**
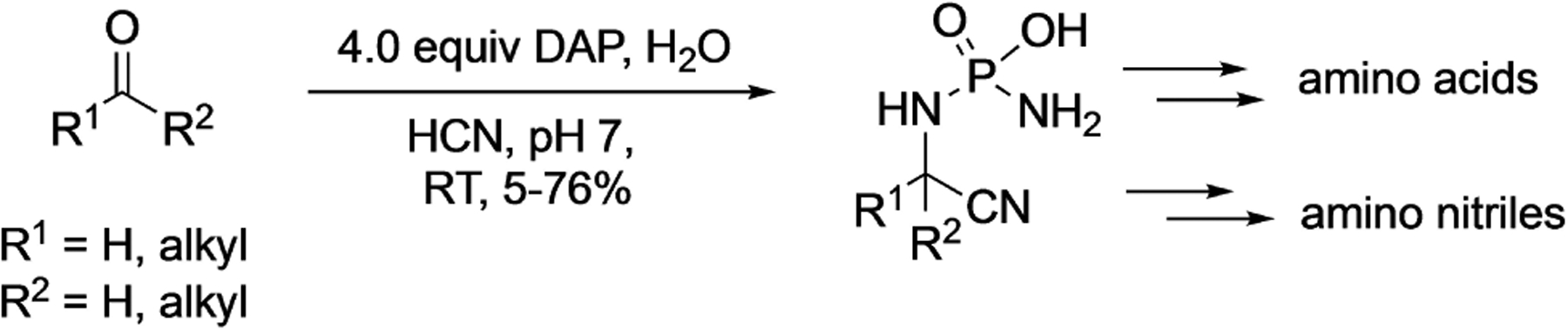
DAP mediated phosphoro-Strecker reaction leading to the formation of phosphoroaminonitriles.

A very different application of DAP was reported in 2019, by Powner’s group who employed DAP as a masked source of ammonia in the Strecker synthesis of amino acids. They observed that the phosphoro-Strecker reaction leads to the formation of phosphoroaminonitriles in high yields starting from ketones and aldehydes, using DAP in the presence of HCN (Scheme 5).^[28]^ The reaction is selective for aldehydes over ketones. These phosphoroaminonitriles were stable at neutral pH and hydrolyzed under extreme acidic or basic conditions to the amino nitriles and amino acids, respectively.

**Scheme 6.**
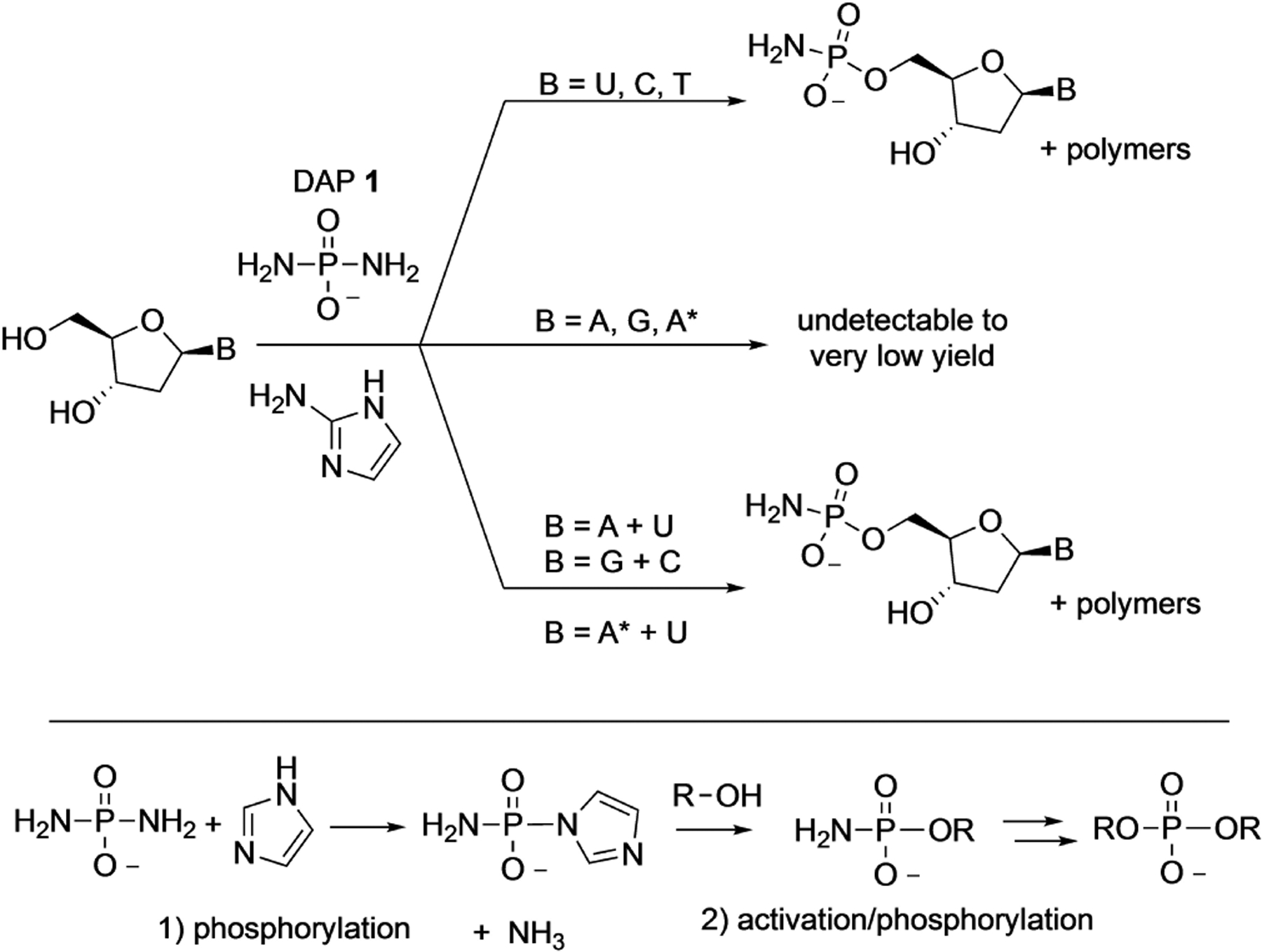
Top: DAP-mediated prebiotic phosphorylation of deoxyribonucleosides with 2-aminoimidazole as the catalyst. Bottom: Mechanism of phosphorylation of DAP that leads to two sequential replacements of the amino groups. T=Thymine, A*=2,6-diaminopurine.

More recently, in 2021, Jimenez et al. reported that the DAP-mediated phosphorylation chemistry under the paste conditions could be extended to deoxynucleosides as well but required change of the activator from imidazole to 2-aminoimidazole.^[29]^ The phosphorylation proceeded primarily at the 5′-position affording the 5′-amidophosphate nucleotides (Scheme 6). Once again, the pyrimidine deoxynucleosides reacted more efficiently than their purine counterparts. In a twist, when purine- and pyrimidine-nucleosides were mixed together in the reaction, the phosphorylation yields of purines increased dramatically. While the exact reasons are not clear, a plausible explanation could be the interactions (hydrogen bonding?) between purines with the pyrimidines leading to enhanced reactivity. It was found that increased temperature (55°C) and wet-dry cycling led to simultaneous formation of (a) oligodeoxynucleotides with predominantly 3′,5′-phosphodiester linkages and (b) 5′-polyphosphorylated species – once again pointing to the versatility of DAP to act both as a phosphorylating and activating agent. Interestingly, pyrophosphate-linked oligonucleotides, which are usually formed in other oligomerization using phosphorimidazolides, were minor components of the reaction products as reported in this work.^[29]^ The mild conditions under which DAP can phosphorylate ribonucleosides to form 2′,3′-cyclophosphates that are activated towards ring-opening reaction makes them suitable for ligation reactions.

**Figure 1.**
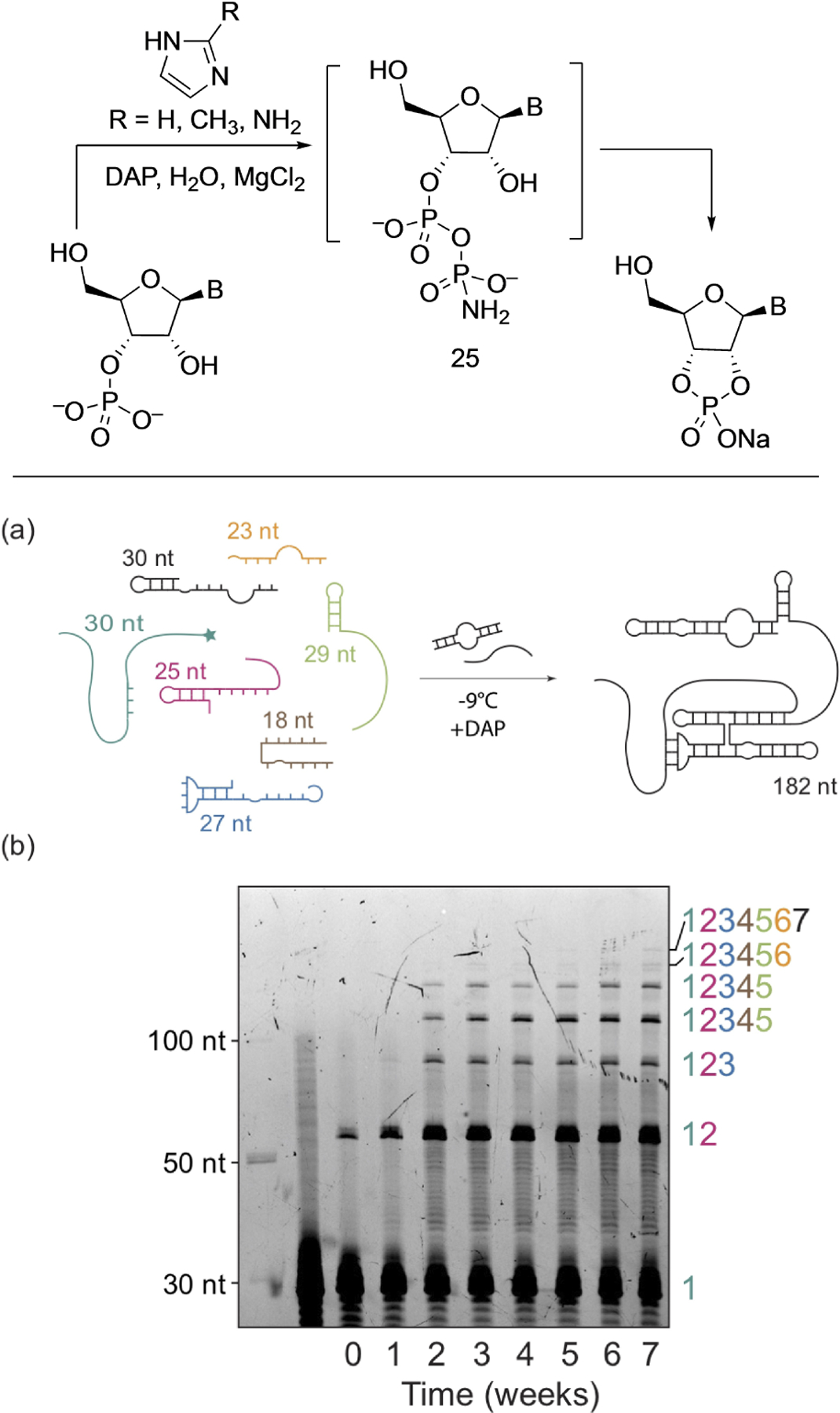
Top: DAP-mediated activation of nucleotide-3′-phosphate forms corresponding 2′,3′-cyclophosphate. Bottom (a–b): This DAP-mediated activation was used towards ligative-assembly of RNA polymerase ribozyme 7 (RPR7) from seven fragments of (<30 nucleotide) oligomers. Figures adapted with permission from Ref. [30]. Copyright 2021 Wiley-VCH.

Thus, Song et al. employed DAP to activate RNA 3′-mono-phosphates to form 2′,3′-cyclophosphates with DAP and imidazole derivatives under frozen water-ice conditions, for a plausible prebiotic activation of RNA suitable for *in situ* hairpin ribozyme ligation catalysis (Figure 1).^[30]^ They demonstrated that DAP in the presence of imidazole activates the 2′ or 3′-phosphorylated ends of RNA oligonucleotides to form the corresponding 2′,3′-cyclophosphate RNA oligonucleotides.

This activation was shown to be long-lived in water-ice, which enabled iterative multiple ligations leading to the assembly of an RNA polymerase ribozyme from seven (≤30 nucleotide) fragments (Figure 1a and 1b). This work showed for the first time that DAP-mediated activation of RNA substrates is compatible with ribozyme catalysis and is superior to the often-used EDC, which is typically used to generate cyclophosphates. EDC, while being kinetically faster in activation and yields in the short term (days), was outperformed by DAP when the reaction proceeded over four weeks. It is also of significance to note that (a) DAP had no noticeable side reactions, unlike EDC which is known to modify the nucleobases and cause side reactions and (b) that DAP is kinetically more stable to hydrolysis while EDC has a considerable shorter half-life in water.^[31]^

The uniqueness of DAP acting as a phosphorylating and activating agent derives from the fact that it has two amino groups already built-in. The first amino group serves as a leaving group enabling the first phosphorylation step while the second amino group on the amidophosphate (on the phosphorylated molecule) is available for the next activation/phosphorylation step (Scheme 6, bottom). This mono-amido-phosphorylated moiety can (a) undergo hydrolysis to form the corresponding phosphate, which can be re-phosphorylated, a process that can be repeated to give rise to polyphosphates, or (b) can be attacked by another nucleophile (such as a nucleoside) to make phosphodiester bonds. However, given the low $\text{p}K_{a_{2}}\;\approx \;1.2$ of the NH_2_ moiety in the amidophosphate derivatives, replacing this NH_2_ with other nucleophiles under ambient conditions is comparatively inefficient. This is affirmed by the low yields and short lengths of oligonucleotides observed in DAP-mediated oligomerizations. At, the same time this ‘lowered’ reactivity of the 5′-amidophosphates could contribute to the lower amounts of pyrophosphate-linked oligonucleotides. Thus, how to address this dichotomy is one area of future research that could significantly affect the future use of DAP in a prebiotic context for non-enzymatic oligomerization.

### 2.2. Application of DAP in organic synthesis

**Scheme 7.**
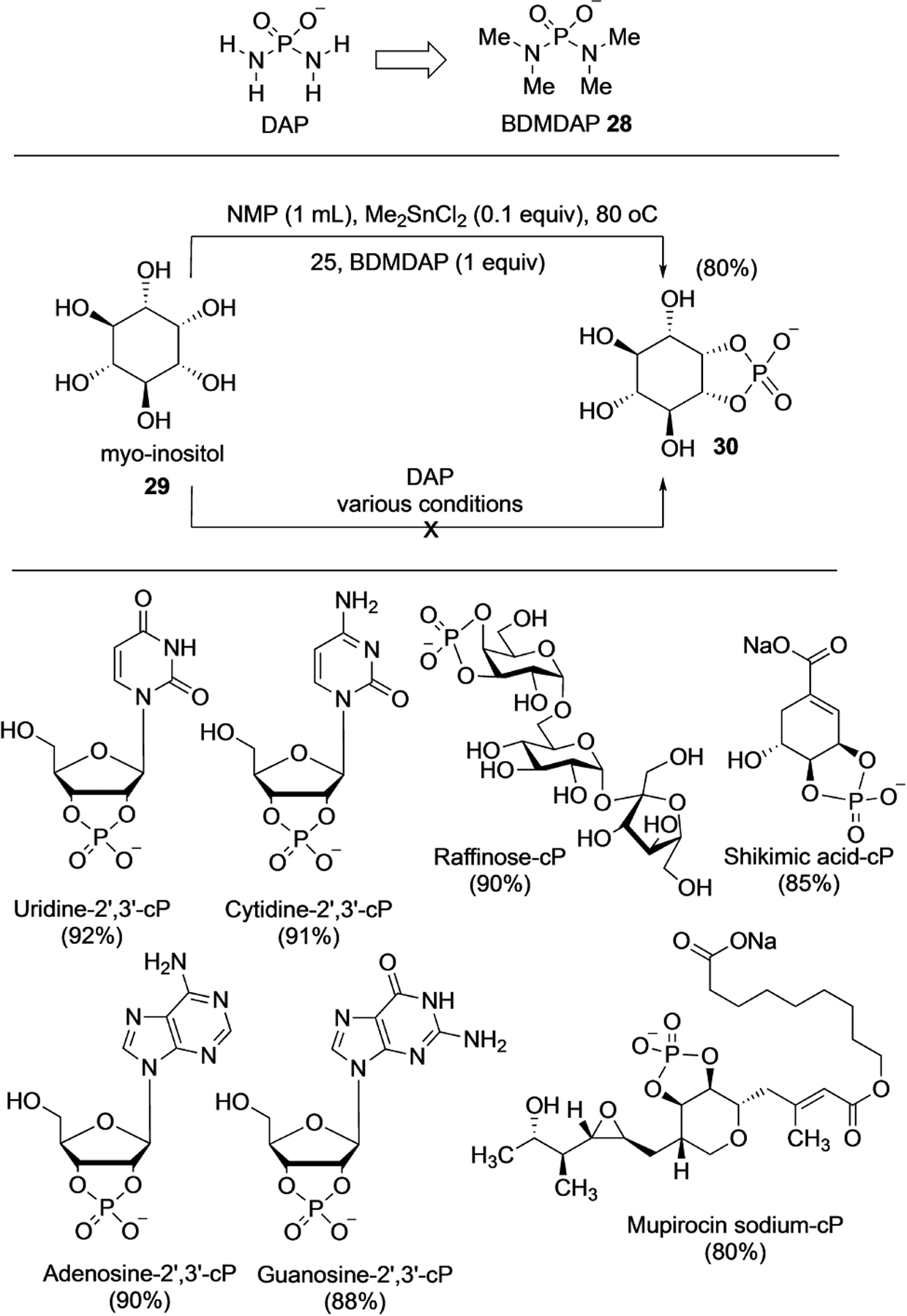
One-pot cyclophosphorylation with BisDiMethylDiAmidoPhosphate (BDMDAP) **28**. Top: Cyclophosphorylation of *myo-*inositol. Bottom: The range of substrates showing diverse tolerance of functional groups. Numbers in bracket refers to isolated yields.

The observation that DAP reacts with *cis*-diols to form the cyclophosphate derivatives, combined with the importance of the cyclophosphate moiety in nature as metabolites and signaling molecules, led Mahipal et al. to develop a one-pot cyclophosphorylating reagent based on DAP-structure and chemistry.^[32]^ Attempts to use DAP itself for cyclophosphorylation in various organic solvents were unsuccessful. With a view to increasing the solubility of DAP in organic solvents, the hydrogens on the 2-amino groups were substituted with methyl groups. This tetramethyl derivative, dubbed BDMDAP **28** (Scheme 7), has a p*K*_a_ of 6.6 compared to DAP’s 5.5, indicating that it would have a higher degree of protonation at neutral pH and act as a better phosphorylating agent.

Systematic investigation of solvents and reaction conditions coupled with Sn(IV) catalysts (which complex with *cis*-diols to increase the nucleophilicity) resulted in the optimal conditions of heating at 80°C with NMP as the solvent. This protocol resulted in a one-step, one-pot, regioselective cyclophosphorylation of the diol and was epitomized by the efficient one-pot synthesis of *myo*-inositol cyclophosphate **30** starting from *myo-*inositol **29** without any protecting group chemistry (Scheme 7). The work also demonstrated the applicability of this method to a wide variety of substrates. From an operational point of view, the simple work-up and purification procedures allowed for routine large-scale operations and access to gram-scale cyclophosphorylated products.

## 3. DAP: Geochemical Availability

The growing role of DAP in prebiotic phosphorylation naturally raises the question about the likelihood of its presence and availability on early Earth.^[12]^ Since PN species in the context of early Earth has not been investigated, it poses a unique challenge and opportunity. One approach that has been taken is to investigate the conversion of reduced forms of phosphorus that is available on early Earth.^[18b]^ An example is the mineral schreibersite, a reduced phosphorus species that have been identified in meteorites and terrestrial Earth.^[18a,33]^ It was reported that schreibersite and its close iron-analogs are corroded by water to produce phosphites and phosphates.^[33a,34]^

**Scheme 8.**
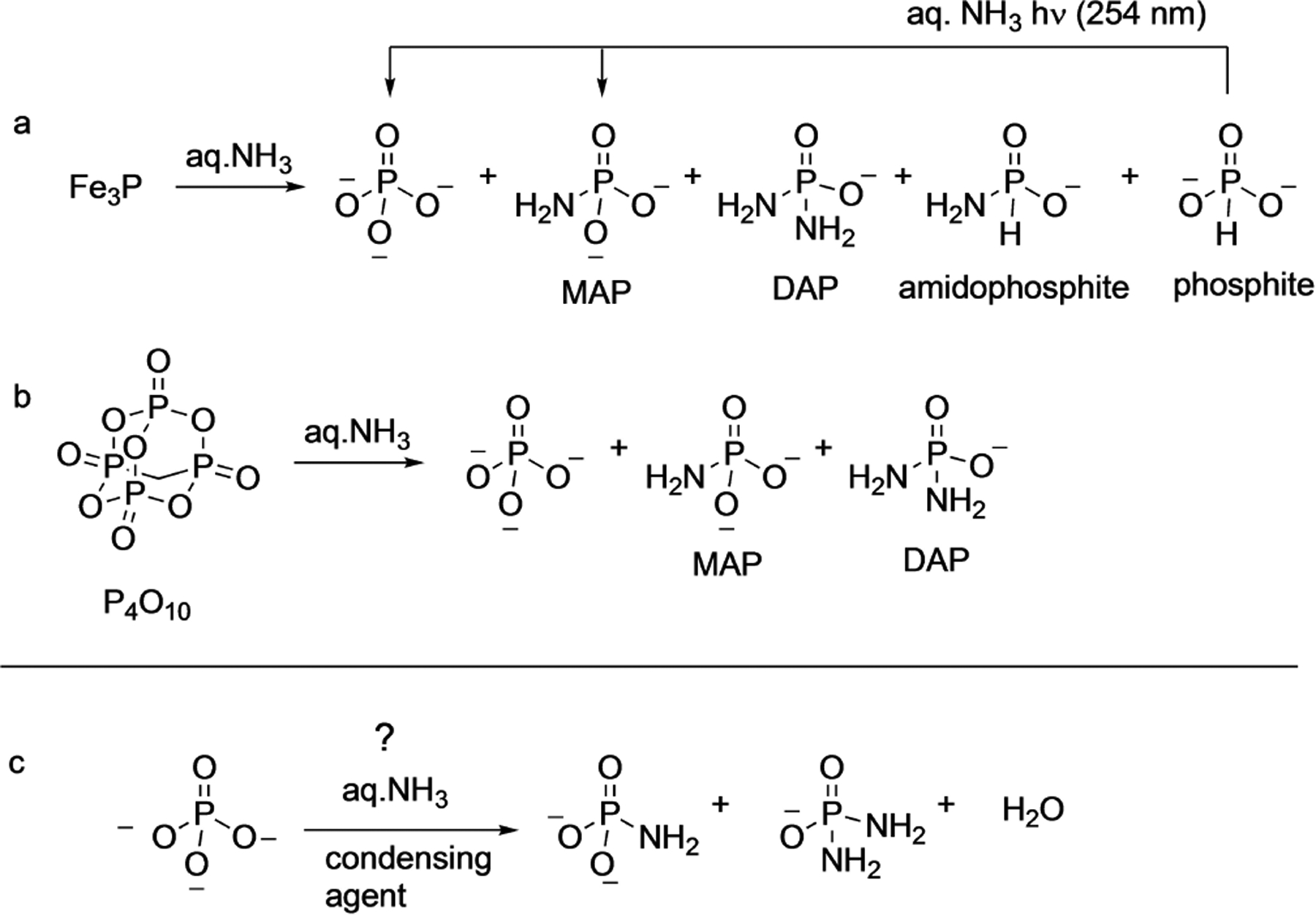
a,b) Plausible prebiotic routes to DAP starting from compounds that were available on early Earth. c) Hypothetical conversion of orthophosphate to amidophosphates using ammonia and condensing agents.

Drawing inspiration from this observation, Gibard et al.^[35]^ in 2019 investigated the corrosion of a schreibersite analog, iron phosphide, in the presence of aqueous ammonia and reported the formation of DAP, MAP along with phosphates and phosphite. They also confirmed the formation of a new species, amidophosphite (Scheme 8a). In the same work, they demonstrated that phosphite can also be converted to MAP by UV irradiation in the presence of ammonia.

The other source for DAP is from the reaction of ammonia with condensed polyphosphates such as the one from cyclotrimetaphosphate that has been pointed out above (Scheme 1b). In that same vein, Gibard et al. showed that P_4_O_10_, phosphorus(V)oxide, can also be converted to DAP and MAP by treatment with aq. ammonia (Scheme 8b).^[35]^ The linear correlation between the amount of PN bonds formed to the amount of NH_3_ in solution suggests that the process – both from schreibersite and condensed phosphates – is fairly efficient. Thus, given the right mix of ammonia in water, it is plausible to produce PN and PO species in the same environment in an early Earth setting. While ammonia availability on early Earth is plausible, whether the concentrations (at least 3% as noted by Gibard et al.) needed for this reaction can be achieved in an early Earth setting needs to be addressed.^[36]^ The authors estimated that ca. 10^15^ to 10^19^ moles of amidophosphates could have been generated over the first billion years on early Earth.

**Figure 2.**
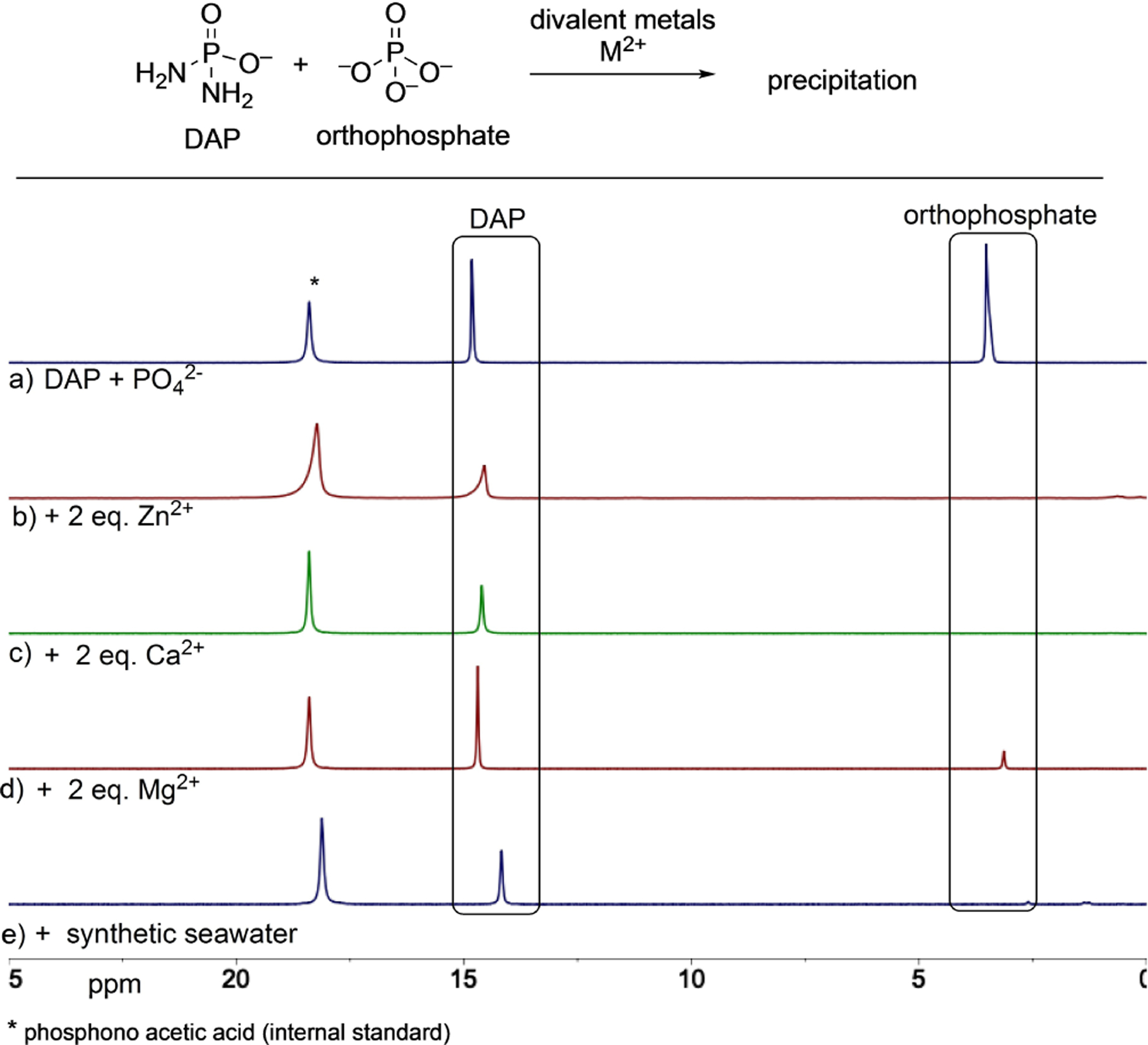
^31^P NMR spectra (b–e) showing the greater availability of DAP versus orthophosphate in solution in the presence of divalent metals – where orthophosphate is precipitated out. Top spectrum (a) is of the standards. Figure adapted with permission from Ref. [35]. Copyright 2019 Wiley-VCH.

A point to be noted when comparing the wide presence of orthophosphates versus the relatively lower prevalence of DAP, is that orthophosphate is precipitated by many divalent metals and thus are not available for reaction in solution. On the other hand, DAP by its virtue of having one less negative charge is less prone to precipitation. This hypothesis was confirmed by Gibard et al. in the same study^[35]^ where they showed that in the presence of divalent metals (Zn^2+^, Mg^2+^, Ca^2+^ and those present in seawater), greater than 50% DAP was still present in solution under conditions where phosphate was completely precipitated out (Figure 2). Thus, on balance, the lower prevalence of DAP (in early Earth scenarios) can be compensated for by its higher solubility/availability and reactivity when compared to orthophosphate.

## 4. Summary and Outlook

### 4.1. DAP: future prospects and challenges

As seen from the above collection of works, DAP is still in its infancy in terms of its phosphorylation potential in the context of prebiotic reactions – as well as synthetic applications. This is in stark contrast to the enormous amount of work with orthophosphates that are not only abundant now, but also ubiquitous on early Earth and in biochemistry. While past investigations, in the context of prebiotic phosphorylation, have focused (logically) on orthophosphates, it is significant to note that almost all of the activation of orthophosphates have used a P–N bond, be it imidazolides or urea. The exceptions are thermal activation to drive off water or the use of other condensing agents.^[37]^ Thus, the ‘hidden’ presence of P–N bonds, used both in prebiotic chemistry and also witnessed in biochemistry, is in congruence with the PN chemistries of DAP.^[12]^

While the advantage of DAP is apparent from (a) its benign activation and reaction conditions to (b) its compatibility with biomolecules (e.g., ribozymes), it *still* does not have the prebiotic credibility of orthophosphates. And that is one of the main challenges that need to be addressed: to demonstrate that amidophosphates can be formed under plausible prebiotic conditions from phosphorus sources that were present on early Earth. For example, can orthophosphate itself be converted to DAP? It is of interest to note that recently, it has been argued that the presence of CO_2_ enables orthophosphate to be available in solution for further processing on the early Earth.^[38]^ Thus, converting orthophosphates to amidophosphates may be a worthy goal to pursue. This could involve a condensation reagent^[37a]^ that would be able to remove water in the process (Scheme 8c). Another avenue to pursue is to check if PN species in minerals from early Earth can be detected. Since PN species have indeed been detected in the interstellar medium,^[39]^ it may be prudent to look for PN species in the context of prebiotic chemistry also on early Earth.^[40]^ In this context, guidance could be taken from the ammonium-phosphate minerals (such as struvite for example) which have been implicated in prebiotic phosphorylation processes,^[17]^ and may have been abundant but fleeting in their existence on early Earth.^[41]^

Irrespective of the prebiotic provenance of DAP, its reactivity in water with organic and biomolecules does open up a new vista for investigating phosphorylation reactions in the context of synthetic organic and bioorganic chemistry. And controlling DAP’s reactivity coupled with enhancing the reactivity of amidophosphates to enable enhanced non-enzymatic oligomerization is not only a challenge but also a worthy goal from many perspectives. In this respect, it may be worthwhile to explore the application potential of DAP – either by itself or making suitable derivatives of DAP.^[32]^ And, all of these future research endeavors^[42]^ will decide the answers to the question raised in the title of this minireview.
